# Rise to modern levels of ocean oxygenation coincided with the Cambrian radiation of animals

**DOI:** 10.1038/ncomms8142

**Published:** 2015-05-18

**Authors:** Xi Chen, Hong-Fei Ling, Derek Vance, Graham A. Shields-Zhou, Maoyan Zhu, Simon W. Poulton, Lawrence M. Och, Shao-Yong Jiang, Da Li, Lorenzo Cremonese, Corey Archer

**Affiliations:** 1 State Key Laboratory for Mineral Deposits Research, Department of Earth Sciences, School of Earth Sciences and Engineering, Nanjing University, 163 Xianlin Avenue, Nanjing 210023, China; 2 Department of Earth Sciences, Institute of Geochemistry and Petrology, ETH, Zürich CH-8092, Switzerland; 3 Department of Earth Sciences, University College London, Gower Street, London WC1E 6BT, UK; 4 State Key Laboratory of Palaeobiology and Stratigraphy, Nanjing Institute of Geology and Palaeontology, Chinese Academy of Sciences, 39 East Beijing Road, Nanjing 210008, China; 5 School of Earth and Environment, University of Leeds, Leeds LS2 9JT, UK; 6 State Key Laboratory of Geological Processes and Mineral Resources, Department of Resource Science and Engineering, Faculty of Earth Resources, China University of Geosciences, Wuhan 430074, China

## Abstract

The early diversification of animals (∼630 Ma), and their development into both motile and macroscopic forms (∼575–565 Ma), has been linked to stepwise increases in the oxygenation of Earth's surface environment. However, establishing such a linkage between oxygen and evolution for the later Cambrian ‘explosion' (540–520 Ma) of new, energy-sapping body plans and behaviours has proved more elusive. Here we present new molybdenum isotope data, which demonstrate that the areal extent of oxygenated bottom waters increased in step with the early Cambrian bioradiation of animals and eukaryotic phytoplankton. Modern-like oxygen levels characterized the ocean at ∼521 Ma for the first time in Earth history. This marks the first establishment of a key environmental factor in modern-like ecosystems, where animals benefit from, and also contribute to, the ‘homeostasis' of marine redox conditions.

## Abstract (web summary)

The Cambrian explosion of biological diversity has been associated with widespread ocean oxygenation, yet early Cambrian ocean redox conditions remain controversial. Here, the authors present a suite of molybdenum isotope data and show that the ocean was oxygenated to modern-like levels by 521 Ma.

## Graphical abstract

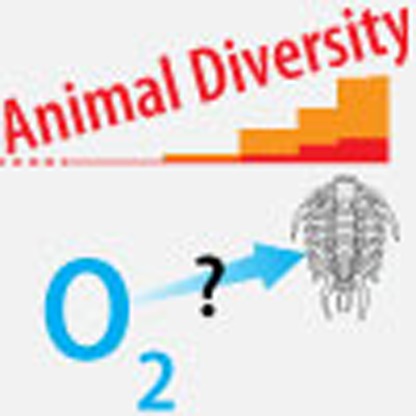

The delay between the origin of animals (∼800 Ma, as inferred from molecular clocks)^1^ and their early diversification in the Ediacaran Period (635–541 Ma)^2^ has been suggested to be due to sluggish oxygenation of the Earth surface^3^,^4^. After the ‘Great Oxidation Event' (GOE^5^) in the early Proterozoic (∼2,400–2,100 Ma), atmospheric 
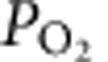
 stayed within ∼0.01 and ∼10% of the present atmospheric level during the mid-Proterozoic (∼2,100–800 Ma (refs 6, 7)). It was only after the termination of the Cryogenian glaciations (∼635 Ma) that oxygen levels in Earth's surface environment began to increase significantly again^8^,^9^. Although the lower estimate of Ediacaran atmospheric 
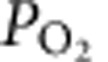
 may surpass the minimum oxygen requirement of animals (<0.1% present atmospheric level)^4^, the deeper ocean likely remained predominantly anoxic^10^,^11^,^12^. As a consequence, O_2_ deficiency^13^, H_2_S toxicity^14^ and a scarcity of trace metal micronutrients (such as Mo, Cu and Zn (ref. 15)) may have continued to limit the ecological distribution of eukaryotes.

Eukaryotes, especially animals and planktonic algae, only began to dominate the marine ecosystem during the ‘Cambrian explosion' of biological diversity (∼520 Ma)^12^,^16^,^17^. Many essential aspects of this biotic event, such as increased animal body size^18^, active locomotion, bioturbation^19^,^20^, carbonate biomineralization^21^, carnivory^13^,^22^ and cropping^17^,^23^,^24^, have been linked to a rise in atmospheric oxygen beyond the minimum requirement of animals, and/or widespread ocean oxygenation. However, redox conditions in the early Cambrian oceans, especially the deep ocean, are still controversial. Some studies suggest widespread oxygenation^13^,^25^,^26^, while others propose a ferruginous (Fe^2+^-rich) or even euxinic (H_2_S-rich) deep marine environment^10^,^27^. Moreover, it has been suggested that oceanic oxygen remained at levels much lower than the modern until the Devonian^28^.

Here we use sedimentary molybdenum (Mo) isotope compositions to trace the evolution of global ocean redox state over this critical period for animal evolution. Global marine redox conditions can be inferred from the sedimentary Mo record because of its redox-sensitive deposition and isotope fractionation mechanisms^9^,^29^,^30^,^31^,^32^. In the modern oxic open oceans, Mo is present as the conservative oxyanion molybdate 
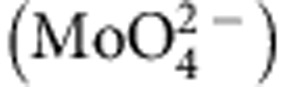
 at relatively high concentrations (its salinity-normalized concentration is ∼107 nmol kg^−1^)^33^. The modern open-ocean seawater (OSW) Mo reservoir is enriched in heavy isotopes (modern *δ*^98/95^Mo_OSW_=+2.34‰, relative to NIST-SRM-3134 (ref. 34)) relative to the dominant input from rivers (*δ*^98/95^Mo_Rivers_=+0.7‰ (ref. 35)). This arises because a major sink for Mo in the modern oceans is the slow adsorption on particulate manganese oxides under widespread oxic conditions, and because this process is accompanied by a −3‰ isotopic fractionation Δ_Sediment–OSW_, that is, *δ*^98/95^Mo_Sediment_−*δ*^98/95^Mo_OSW_=−3‰ (refs 29, 36)). When bottom-water dissolved oxygen is low or absent, H_2_S may be present in pore waters of organic-rich reducing sediments (on some continental margins) or in the water column (for example, Black Sea and regions of intense upwelling). Under these conditions, Mo deposition can be accelerated by one to two orders of magnitude^37^,^38^, leading to smaller isotopic fractionations Δ_Sediment–OSW_=0 to −0.7‰ (ref. 39)) due to more quantitative sequestration from the dissolved phase in these settings. An expansion of such sulphidic conditions will therefore cause the *δ*^98/95^Mo value of seawater to decrease towards the riverine input value. Seawater *δ*^98/95^Mo will most likely be recorded in sediments deposited under euxinic conditions because of quantitative removal of aqueous Mo. However, importantly, measured *δ*^98/95^Mo values of sediments provide a minimum constraint on contemporaneous seawater isotopic composition because all known sedimentary Mo sinks record *δ*^98/95^Mo≤seawater^28^. We determined Mo concentrations and isotope compositions (see Methods) of well-preserved organic-rich marine sediments of Cryogenian to early Cambrian age. We also assess local water column redox conditions using iron speciation (see Methods) in the same sediments. We find that the tempo of ocean oxygenation, as reconstructed by the Mo isotope and concentration profiles, was in step with the early Cambrian bioradiation.

## Results

### Geologic setting and samples

Late Neoproterozoic to Cambrian successions are well developed in South China. They are fossiliferous, with several unique biotas in various environments (Supplementary Note 1) and provide one of the best candidates for constraining the co-evolution of marine oxygenation and life^9^,^14^,^40^. We collected black shales and organic-rich cherts from shallow-to-deep water successions on the south-eastern margin of the Yangtze platform (Supplementary Figs 1 and 2). The ages of our samples span the mid-Cryogenian to the early Cambrian (∼660–520 Ma). Ediacaran and early Cambrian successions in the Yangtze Gorges area represent shallow-water facies, while deeper water facies occur in northwestern Hunan and southern Anhui provinces (see Supplementary Note 1).

### Mo isotopes

**Figure 1:**
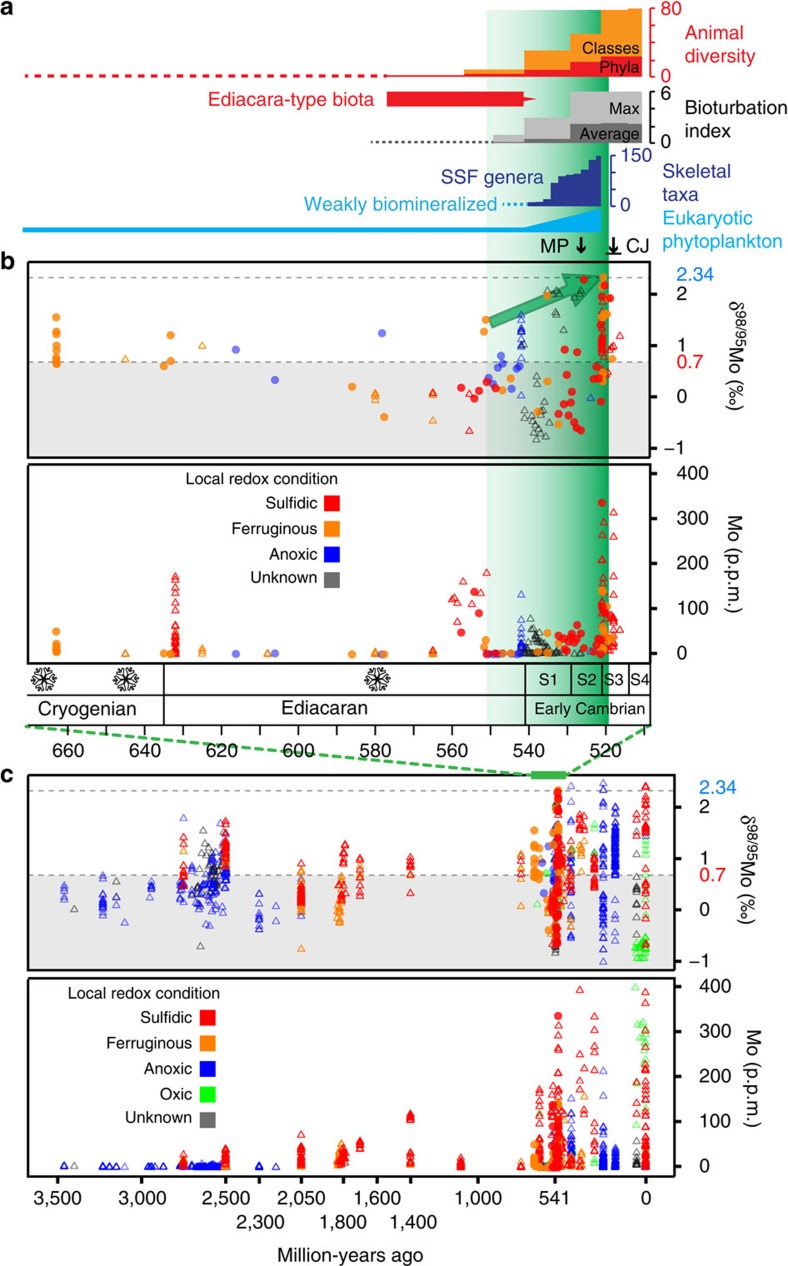
Compilation of Mo data together with biodiversity and degree of bioturbation during the Ediacaran–Cambrian transition. (**a**) Bioturbation indices and diversities of animals, skeletal taxa and eukaryotic phytoplankton^1^,^20^,^59^. MP: mesozooplankton appeared^17^; CJ: the Chengjiang Lagerstätte. (**b**) Mo data from the mid-Cryogenian to the early Cambrian. In the timescale, three major glaciations are marked as snowflakes, S1 to S4 denote the first four Cambrian stages. The colour of the data points denotes local redox: sulphidic (Fe_Py_/Fe_HR_>0.7, red), ferruginous (Fe_Py_/Fe_HR_<0.7, orange), anoxic (blue, when Fe speciation data are not available, Fe/Al>0.5, trace metal enrichments and other sedimentary characteristics are used to discriminate anoxic conditions) and unknown (grey, no above mentioned data are available, also included are typical carbonates and phosphates, to which Fe-S-C systematics redox proxies cannot easily be applied). The dashed lines mark the average *δ*^98/95^Mo value of modern seawater (+2.34‰) and the riverine input (+0.7‰). Data sources, filled circles: this study; open triangles: published data. Mo concentrations of samples from the early Cambrian Ni–Mo ore layer are not shown because of their exceptional enrichment in Mo (in the percent range). The green arrow marks the rising maximal *δ*^98/95^Mo values. The graded green shading in **a**,**b** denotes postulated oxygenation of the ocean from the late Ediacaran to the early Cambrian. (**c**) Mo data from the Palaeoarchaean to present (Supplementary Data 1). Symbols as in **b**, and green colour stands for oxic condition (Fe_HR_/Fe_T_<0.38 and/or Fe/Al<0.5).

Our Mo concentration and isotopic data, together with published data from the late Archaean to early Cambrian, are compiled in Supplementary Data 1 and shown in Fig. 1 and Supplementary Fig. 2. After several moderate peaks (+1.5‰ to +1.7‰) without a distinct increase in Mo abundance in the late Archaean (∼2,500–2,750 Ma)^41^,^42^,^43^,^44^, presaging the GOE, *δ*^98/95^Mo remained at low levels (<+1.3‰) from the late Palaeoproterozoic (∼2,300 Ma) to the mid-Neoproterozoic (∼750 Ma)^28^,^30^,^41^,^45^,^46^,^47^,^48^. In the late Neoproterozoic, higher *δ*^98/95^Mo values (up to +1.6‰) are tied to increased Mo abundance in the aftermath of the three major glaciations (Sturtian, Marinoan and Gaskiers), possibly linked to oxygenation events driven by high nutrient inputs from enhanced terrestrial weathering^6^,^8^. *δ*^98/95^Mo values approached >+1.5‰ around 550 Ma, and then reached +2‰ for the first time in Earth history during the earliest Cambrian (∼535 Ma, mid-Fortunian Stage). After that, coinciding with the first occurrence of trilobites and nearly all animal clades in the major phase of the ‘Cambrian explosion', both high *δ*^98/95^Mo values (near +2.3‰) and high Mo concentrations (>100 p.p.m.) are found in samples from different locations, and cluster around the Cambrian Stage 2/3 boundary (521 Ma). There are four sulphidic samples out of eight samples having high *δ*^98/95^Mo values (>+1.9‰) between ∼525 and ∼520 Ma. Although the other half of these eight samples were deposited under Fe-rich conditions (note that these samples still contain appreciable pyrite, with Fe_Py_/Fe_HR_>0.3 to ∼0.5), we emphasise that the sediments analysed likely record *δ*^98/95^Mo close to that of contemporaneous seawater (for the sulphidic black shales) or a minimum value for seawater (for the ferruginous black shales).

## Discussion

Our data provide an estimate for the lower limit of coeval seawater Mo isotopic composition and document the fact that *δ*^98/95^Mo_OSW_ rose to a level higher than ever before during the early Cambrian, peaking at modern levels (∼+2.3‰) at ∼520 Ma. The rarity of black shales deposited under fully euxinic conditions makes a continuous record of the precise *δ*^98/95^Mo of seawater difficult to obtain. However, where sulphidic black shale data (or phosphatic data, which have been suggested also to record seawater values^25^) are available, their *δ*^98/95^Mo data are lower than those at 520 Ma—that is, +1.2‰ for 750 Ma (ref. 49), +0.2‰ for ∼551 Ma (ref. 50 and our data), and +2‰ for phosphorites at ∼535 Ma (ref. 25). The early Cambrian marine oxygenation event delineated by maximal *δ*^98/95^Mo values in black shales is consistent with other geochemical records, such as iron speciation and uranium concentrations (Supplementary Fig. 3). Canfield *et al.*^10^ established an iron speciation database and found that anoxic ferruginous deep oceans were widespread and persistent during later Neoproterozoic times. Li *et al.*^14^ analysed the iron speciation systematics of samples obtained from a shore-to-basin transect and proposed that sulphidic zones were sandwiched between ferruginous waters on continental margins through the Ediacaran Period. Owing to geochemical similarities with Mo, U concentrations in sediments can also reflect the redox state of the global ocean^26^, although the onset of U enrichment in sediments requires less reducing conditions than that of Mo^51^. Hence, U concentrations in sediments deposited under both sulphidic and anoxic non-sulphidic conditions can indicate the size of the ocean uranium reservoir, which is proportional to the level of ocean oxygenation. Available data show that U concentrations in anoxic sediments are generally higher for the early Cambrian than for the Ediacaran, and also peaked at ∼520 Ma (see Supplementary Fig. 3).

**Figure 2:**
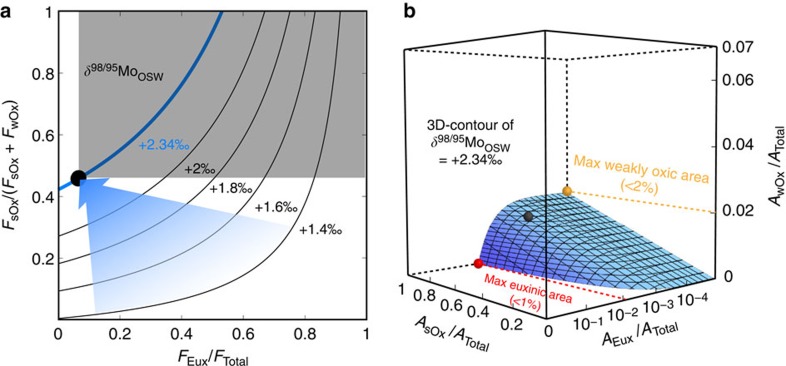
Model of open ocean seawater *δ*^98/95^Mo (*δ*^98/95^Mo_OSW_) in response to different proportions of Mo sinks. (**a**) Contours of *δ*^98/95^Mo_OSW_ as a function of the relative sizes of the euxinic (*F*_Eux_), weakly oxic (*F*_wOx_) and strongly oxic (*F*_sOx_) sinks, assuming that the Mo cycle is in steady state. The black dot represents the modern budget. The upper shaded area would represent coupled expansion of euxinia and the strongly oxic condition relative to the weakly oxic condition, which is unrealistic^28^. The blue arrow denotes a possible oxygenation trend of the ocean, dictated by the Mo data and the model, from the Neoproterozoic to the early Cambrian. (**b**) A 3D contour surface of *δ*^98/95^Mo_OSW_=+2.34‰ as a function of areal (*A*) fractions of the above redox settings, assuming that the Mo cycle is in steady state. The black dot represents modern redox area distributions; red and yellow dots represent maximum euxinic (1%) and weakly oxic (2%) areas, respectively.

Peak *δ*^98/95^Mo values indicate that oxygenation of the ocean reached modern-like levels for the first time in Earth history at ∼520 Ma. The *δ*^98/95^Mo_OSW_ value of the ocean can attain such high values under two alternative scenarios: either oxic waters overwhelmingly dominated the global seafloor in a steady-state Mo cycle, or widespread mildly euxinic waters suddenly consumed the ocean Mo reservoir in a catastrophic hydrogen sulphide-release event^27^. The latter scenario is inconsistent with the high *δ*^98/95^Mo values found in this study for black shales both below and above the peak Mo concentration layer (that is, the Ni–Mo-enriched layer, see Supplementary Note 1), while the peak itself has only intermediate *δ*^98/95^Mo values (<+1.4‰ (refs 52, 53)). As increases in *δ*^98/95^Mo and U concentrations both exhibit a long-term trend through the early Cambrian, we apply an improved steady-state mass balance model (see Methods and below), which demonstrates that *δ*^98/95^Mo values of *ca.* +2.3‰ indicate an unprecedentedly high level of marine oxygenation. We divide the Mo sinks into three types with increasing Mo accumulation rates and decreasing Mo isotope fractionation^37^,^38^: a sink under strongly oxic water (denoted sOx, where O_2_ penetrates >1 cm below the sediment–water interface, average Mo isotope fractionation Δ_sOx–OSW_=−2.95‰ (ref. 29)); a sink under weakly oxic water (wOx, low O_2_ in bottom waters and H_2_S exists in organic-rich shallow sediments, average Mo isotope fractionation Δ_wOx–OSW_=−0.7‰ (ref. 39)); and a sink under euxinic water (H_2_S is present in bottom waters, average Mo isotope fractionation Δ_Eux–OSW_=−0.5‰ (ref. 28)). We also note that in the modern oceans there are large areas of *moderately* oxic seafloor (mOx, ∼14% (ref. 38)), where the sulphidic zone is deep in the sediment column and the bottom-water O_2_ concentration is higher than or comparable to the weakly oxic condition. In these settings Mn oxide-related Mo is quantitatively remobilized from shallow sediments and released back to seawater^37^,^38^,^54^. Such areas are important but not relevant to the mass balance model since they are neither a sink nor a source for Mo. Our modelling results (Fig. 2) suggest that Mo removal under oxygenated bottom waters (including both strongly and weakly oxic conditions) must account for more than 94% of the total Mo sink when *δ*^98/95^Mo_OSW_ reaches modern-like levels (+2.3‰), but can be as low as 33% when *δ*^98/95^Mo_OSW_ is +1.6‰, which is the highest observed value for the Proterozoic. The modern-like *δ*^98/95^Mo_OSW_ value also requires that the strongly oxic sink accounts for more than 42% of the total oxic sink, while a *δ*^98/95^Mo_OSW_ value of +1.6‰ would allow weakly oxic sink to account for up to 91% of the total oxic sink.

The areal proportions of the three redox conditions are further explored through incorporating Mo accumulation rates (see Methods). There is a wide range in estimated Mo accumulation rates for modern euxinic settings (*F'*_Eux_), from 12,000 μg m^−2^ yr^−1^ (ref. 32) to 4,800 μg m^−2^ yr^−1^ (ref. 38), and we here choose the lower rate to avoid overestimating both the difference between euxinic and oxic sinks, and the oxic area fraction. To maintain not only mass but also isotope balance of the modern oceanic Mo cycle, a *F*'_sOx_ value of 40 μg m^−2^ yr^−1^, which is higher than previous estimate of 27.5 μg m^−2^ yr^−1^ (ref. 38), is required. Our modelling results (Fig. 2) indicate that a *δ*^98/95^Mo_OSW_ value of +2.3‰ requires a limited extent of both euxinic (<1%) and weakly oxic (<2%) areas. Therefore, strongly and moderately oxic areas, with relatively high O_2_ concentrations in bottom waters, must have covered >97% of the seafloor at ∼521 Ma, at least episodically, in comparison with a coverage of 80% to satisfy a *δ*^98/95^Mo_OSW_ value of +1.6‰. This oxygenation event was broadly contemporaneous with the Cambrian bioradiation (Fig. 1).

The early Cambrian bioradiation is characterized by the emergence of nearly all major bilaterian body plans^1^. This developmental/morphological complexity is a long-term consequence of eukaryotic multicellularity, which had originated by the late Mesoproterozoic (∼1,200 Ma) and which diversified during the Ediacaran^12^,^17^,^55^. However, the Ediacara-type biota (∼575–541 Ma) was dominated by soft-bodied sessile epibenthic osmotrophs, and there is no unambiguous evidence for motile bilaterians^56^,^57^, except towards the very end of the Ediacaran^58^. From the terminal Ediacaran to the earliest Cambrian (∼580–529 Ma), shallow bioturbation^20^ and phosphatic or aragonitic small shelly fossils characterize the initial phase of the ‘Cambrian explosion'^59^. During Cambrian Stage 2 (∼529–521 Ma), diverse deeper burrows and complex trace fossils record active bioturbation of sediments and mark the so-called ‘substrate/agronomic revolution'^20^, while small shelly fossils further diversified and calcitic taxa began to appear^59^,^60^. Nearly all bilaterian body plans had appeared by early Cambrian Stage 3 (ref. 1), the major phase of the ‘Cambrian explosion'.

The apparently abrupt appearance of large, motile and diverse animal forms, following a prolonged and obscure history of phylogenetic evolution, has frequently been explained by changes in the physical environment, especially redox conditions^1^,^13^, although this conclusion is not without controversy^4^. Our data clearly indicate a spatial waning of anoxia (including euxinic and non-sulphidic conditions) in the early Cambrian ocean, which may have been a pre-condition for the transition to a modern marine ecosystem supporting diversified animals. If anoxia were widespread, even though early animals may have inhabited patchy oxic environments, intrusion/upwelling of anoxic water would have stifled their success^14^,^61^. Diminished euxinia may also have relieved the Proterozoic trace metal micronutrient crisis^15^.

Our data also indicate unprecedentedly widespread oxygenation in the early Cambrian ocean. The expansion of strongly and moderately oxic seafloor from <80% during the Precambrian to >97% during the early Cambrian may have provided a significant increase in the size of habitable space for animals. Animals favour continental marginal areas that have abundant nutrients but are also prone to anoxia because of high primary productivity and the consequent high O_2_ demand. When >97% of the seafloor was well oxygenated, there must have been vast space (>60% of the continental margins) for animals to flourish, given that modern continental margins occupy ∼7% of the world's seafloor. This expansion of oxygenated seafloor took place against the backdrop of the rising sea level in the early Cambrian^62^, creating numerous new oxygenated ocean margin settings and liberating animals that possibly suffered from fluctuating redox conditions on the continental shelf during Ediacaran time^14^,^61^. Moreover, importantly, the expansion of oxic seafloor to a modern extent may imply near-modern average marine oxygen concentrations. Although animals, especially those of sponge-grade and meiofauna, could survive at modest oxygen levels^4^,^63^, the large size and ecological dominance of the characteristic Cambrian fauna could not have developed without abundant oxygen. Metabolically expensive behaviours, such as active locomotion, bioturbation and muscular carnivory, require a high oxygen consumption rate for efficient aerobic respiration^13^,^20^. Moreover, oxygenation would have favoured aerobic over anaerobic respiration in the deep ocean, resulting in a decrease in alkalinity and thus suppressing carbonate deposition on/in the seafloor^21^. This, consequently, would have increased surface ocean carbonate supersaturation, which may have reduced the physiological cost of carbonate skeleton construction and facilitated the evolutionary arms race^13^.

Animal ecosystem engineers may also have contributed to ocean oxygenation^12^,^17^. The early originating suspension-feeding sponges had low O_2_ requirements^63^, and may have helped consume the large dissolved organic carbon reservoir that acted as a major redox buffer in the Proterozoic ocean^12^. Planktonic animals and algae^64^ diversified nearly simultaneously in the early Cambrian, and likely enhanced the efficiency of the biological pump^12^,^17^,^65^, lowering oxygen demand in the water column. Once benthic motile animals became widespread, they deepened O_2_ penetration depth through bioturbation, which helped P-retention and in turn limited primary production and stabilized marine oxygenation^12^.

In sum, a pervasively well-oxygenated ocean would have played a critical role in the ‘Cambrian explosion', during which newly evolved animals and ecosystems affected both carbon and nutrient cycling, first facilitating and then stabilizing more widespread oxygenation in the world's oceans^12^,^17^. Through a combination of high-resolution palaeoenvironmental and palaeobiological studies, we are reaching a more comprehensive understanding of the complex, dynamic interactions and feedbacks that helped to bring modern-like ecosystems into being.

## Methods

### Samples

Fresh rock samples were powdered using an agate mill. Trace metal and Mo isotope analyses were carried out with an Element II ICP-MS and a Neptune MC-ICP-MS, respectively, at the University of Bristol. Samples were digested by standard HF-HNO_3_-HCl methods at 180 °C for >72 h.

### Mo isotope composition

Mass spectrometry and mass bias correction of Mo isotope data by the double spike technique were carried out as previously described^35^. Mo isotope data are reported using the *δ* notation, relative to the Mo standard NIST-SRM-3134 (with its *δ*^98/95^Mo value of +0.25‰ (refs 34, 66)) where


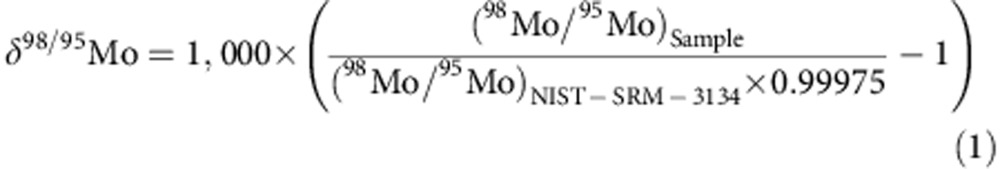


The internal precision of Mo isotopic measurements ranges between 0.02‰ and 0.05‰ (2SE of 30 integrations) for all our samples. The external reproducibility of a series of replicates (entire procedure from digestion of powder onwards) of a black shale sample is 0.02‰ (2SD, six replicates).

In this study only samples with Mo-enrichment factor (Mo_EF_, which is defined as the ratio of ([Mo]/[Al])_Sample_ to ([Mo]/[Al])_Crust_) more than two are plotted, to ensure that only the isotopic composition of authigenic Mo is considered. We did calculations to correct the *δ*^98/95^Mo values for detrital Mo contribution^39^, assuming that the total Mo is a mixture of the detrital (*δ*^98/95^Mo=0‰) and authigenic Mo. Samples having a difference >0.2‰ in *δ*^98/95^Mo between the measured and the corrected values were removed. All Mo isotope data are presented without detrital Mo correction.

### Iron speciation

We further filtered our samples to include only those deposited under anoxic conditions. We accessed the local redox conditions by iron speciation. Iron in carbonate (Fe_Carb_), oxides (Fe_Ox_) and magnetite (Fe_Mag_) was sequentially extracted at the Newcastle University using the method described in ref. 67. Iron in pyrite (Fe_Py_) is calculated from pyrite sulphur measured by the chromium reduction method, given a stoichiometry for pyrite of FeS_2_. Total organic carbon (TOC) contents were measured at the University College London using a Leco C/S analyser after acidification with 6 N HCl.

In general, under an anoxic (O_2_-free, with or without H_2_S) water column, highly reactive Fe (Fe_HR_) usually constitutes more than 38% of total Fe (Fe_T_) because of syngenetic formation of either iron sulphides in sulphidic waters or non-sulphidized minerals (Fe carbonate, Fe^III^ oxides or magnetite) in ferruginous waters. A Fe_Py_/Fe_HR_ ratio of 0.7 (ref. 11) is used to discriminate between ferruginous (<0.7) and sulphidic (>0.7) anoxia in the water column.

### Mass balance model of Mo cycle

The parameters used in the model are listed in the Supplementary Table 1. We divide the Mo sinks into a strongly oxic sink (*F*_sOx_, bottom-water dissolved oxygen >10 μM, where O_2_ penetrates >1 cm below the sediment–water interface), a weakly oxic sink (*F*_wOx_, bottom-water dissolved oxygen <10 μM, with a shallow sulphidic zone in the reducing sediments) and a euxinic sink (*F*_Eux_, H_2_S is present in bottom water)^37^,^38^. The moderately oxic seafloor, with a deep sulphidic zone in the sediment column, is not relevant to the global Mo budget because Mn oxide-related Mo is quantitatively released back into the water column during reduction. Because Δ_Eux–OSW_ (=*δ*^98/95^Mo_Eux_−δ^98/95^Mo_OSW_) approaches 0 only if bottom-water [H_2_S]_aq_ is greater than 11 μM, an average fractionation of −0.5‰ (Δ_Eux−OSW_) is used for the euxinic sink^28^. The Mo isotope composition of open ocean seawater (*δ*^98/95^Mo_OSW_) is determined by the fractions of the fluxes to sediments in the three redox settings (*f*_Eux_, *f*_wOx_ and *f*_sOx_) with the assumption of steady-state, and setting the input to the ocean at the modern value of the riverine flux:


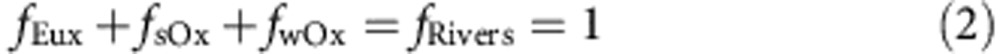


Isotope mass balance is described by:


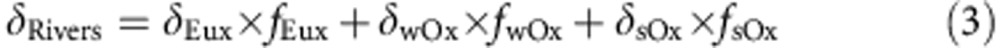


where each *δ*_Output_ equals (*δ*^98/95^Mo_OSW_+Δ_Output_).

We define:


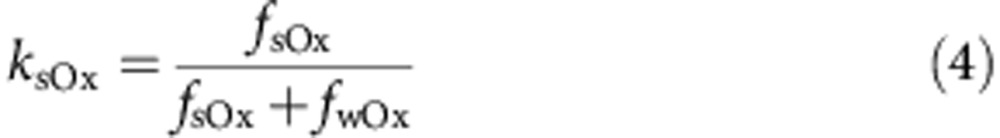


and solve the above three equations to get the *δ*^98/95^Mo_OSW_ as a function of *k*_sOx_ and *f*_Eux_, and then plot the contours of *δ*^98/95^Mo_OSW_ in Fig. 2.

The Mo output rates (*F'*_Output_, g m^−2^ yr^−1^) in various redox settings are assumed to be controlled by first-order kinetics with respect to the coeval Mo reservoir in the open ocean (*R*), that is, each *F'*_Output_=*F'*_Output0_ × *R*/*R*_0_ (subscript 0 denotes the modern value). Replacing each *f*_Output_ (=*F*_Output_/*F*_Rivers_) in the isotope mass balance equation (3) with:


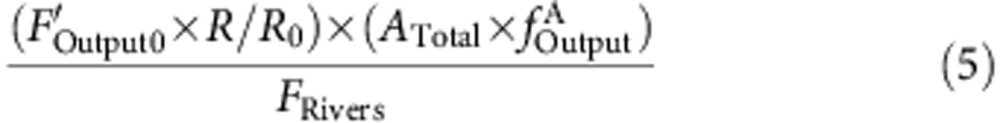


together with the mass balance equation (2), we can get the *δ*^98/95^Mo_OSW_ as a function of areal fractions of the three redox settings 
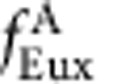
, 
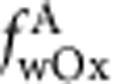
 and 
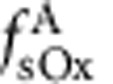
.

In our sensitivity analyses (see Supplementary Fig. 4) we first investigated the impact of setting both Δ_Eux−OSW_ and *δ*_Rivers_ to zero. This results in even smaller areas of euxinia and greater predominance of oxic bottom waters for modern-like *δ*^98/95^Mo_OSW_, including a major contribution of strongly oxic conditions.

In modern weakly oxic settings, although the uppermost few centimetres of sediments show heterogeneous Δ_Sediment–OSW_ ranging from −1.2‰ to −0.2‰, the Δ_Sediment–OSW_ of deep sediments converge on a common value of −0.7‰, which more likely represents the true average Δ_wOx–OSW_ over long timescales^39^. However, we further investigated the impact of uncertainty in Δ_wOx–OSW_. A smaller fractionation, such as Δ_wOx–OSW_ of −0.2‰, results in a similar situation to the previous sensitivity analyses, that is, even greater contributions are required from oxic sinks for modern-like *δ*^98/95^Mo_OSW_. Although Δ_wOx–OSW_ of −1.2‰ would allow smaller contributions from oxic sinks, modern-like *δ*^98/95^Mo_OSW_ still requires/reflects a predominance of oxic sinks.

## Additional information

**How to cite this article:** Chen, X. *et al.* Rise to modern levels of ocean oxygenation coincided with the Cambrian radiation of animals. *Nat. Commun.* 6:7142 doi: 10.1038/ncomms8142 (2015).

## Supplementary Material

Supplementary Information Supplementary Figures 1-4, Supplementary Table 1, Supplementary Note 1 and Supplementary References

Supplementary Data 1 Mo concentration and isotope composition for all samples analysed and compiled in this study. All Mo isotope data are renormalised relative to NIST-SRM-3134 molybdenum standard according to Goldberg et al. 2013.
