# Supplementary material for: Rise to modern levels of ocean oxygenation coincided with the Cambrian radiation of animals
Source: Nat Commun. 2015 May 18;6:7142. doi: 10.1038/ncomms8142 (PMC4479002; doi:10.1038/ncomms8142)
Supplement: Supplementary Information — Supplementary Figures 1-4, Supplementary Table 1, Supplementary Note 1 and Supplementary References [file ncomms8142-s1.pdf]

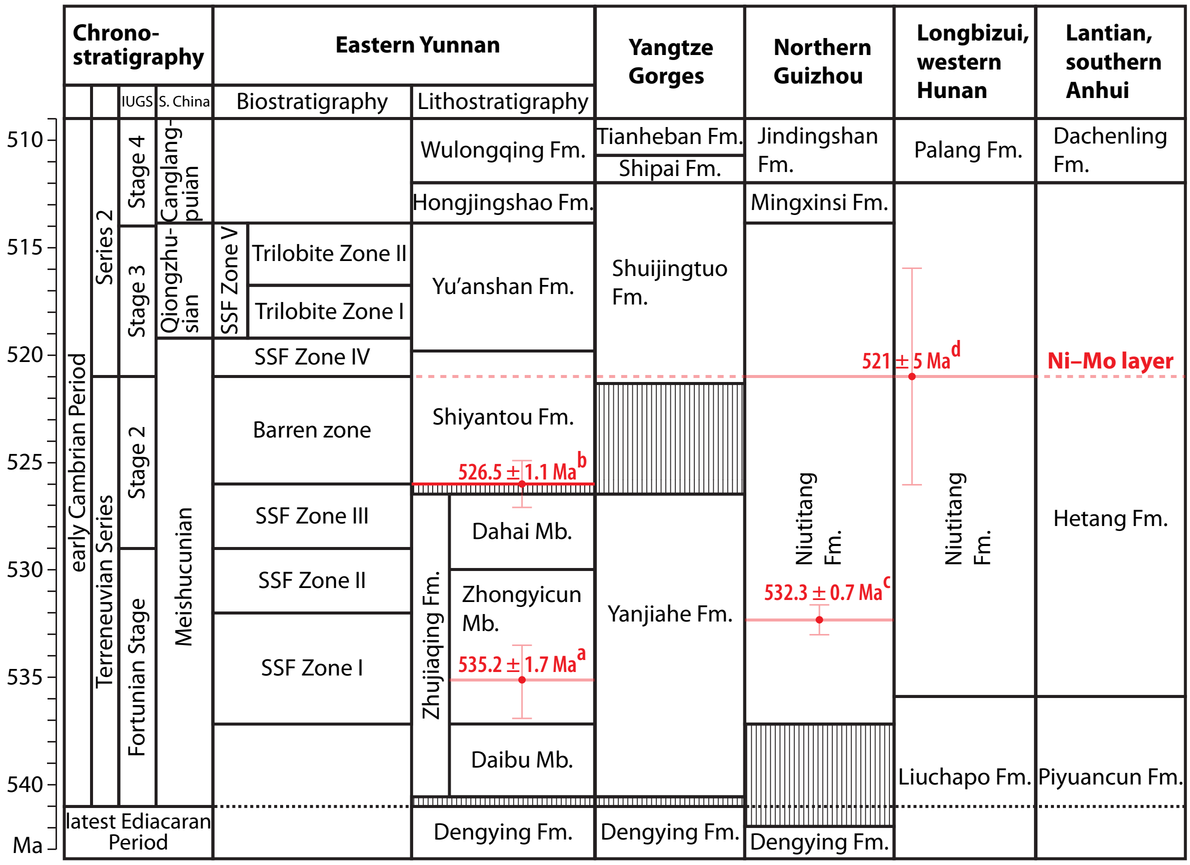

Supplementary Figure 1 | Lower Cambrian chronostratigraphy ratified by IUGS[1] and coeval regional division schemes in South China, as well as stratigraphic correlation in the relevant sections from South China. SSF zones are established in eastern Yunnan[2–5]. The Ediacaran Dengying Formation (mainly dolostone), deposited in shallow shelf settings, can be correlated with the Liuchapo and Piyuancun Formations (mainly cherty rocks) deposited in deep slope/basin settings[6]. While deposition across the Ediacaran/Cambrian was continuous in deeper settings, hiatuses with different extents between the Ediacaran and the Cambrian have been recognised in shallower settings[5]. Based on FAD of SSFs and chemostratigraphic data, the Ediacaran–Cambrian boundary has been placed within the top of the Dengying Formation or in the hiatus above it (e.g. refs 2,7) in shallow-water sections, and within the top part of the Liuchapo and Piyuancun Formations in deep-water sections (Longbizui and Lantian)[8,9]. There is a Ni–Mo-enriched layer that occurs synchronously in the early Cambrian black shale, distributed in a 1600-km-long belt along the southern margin of the Yangtze platform[10–14]. Radiometric ages: a. The middle (bed 5) Zhongyicun Member[15]; b. The bottom of Shiyantou Formation[16]; c. The lowermost phosphate nodule-bearing black shale of the Niutitang Formation[17]; d. The Ni–Mo layer in the Niutitang Formation[18].

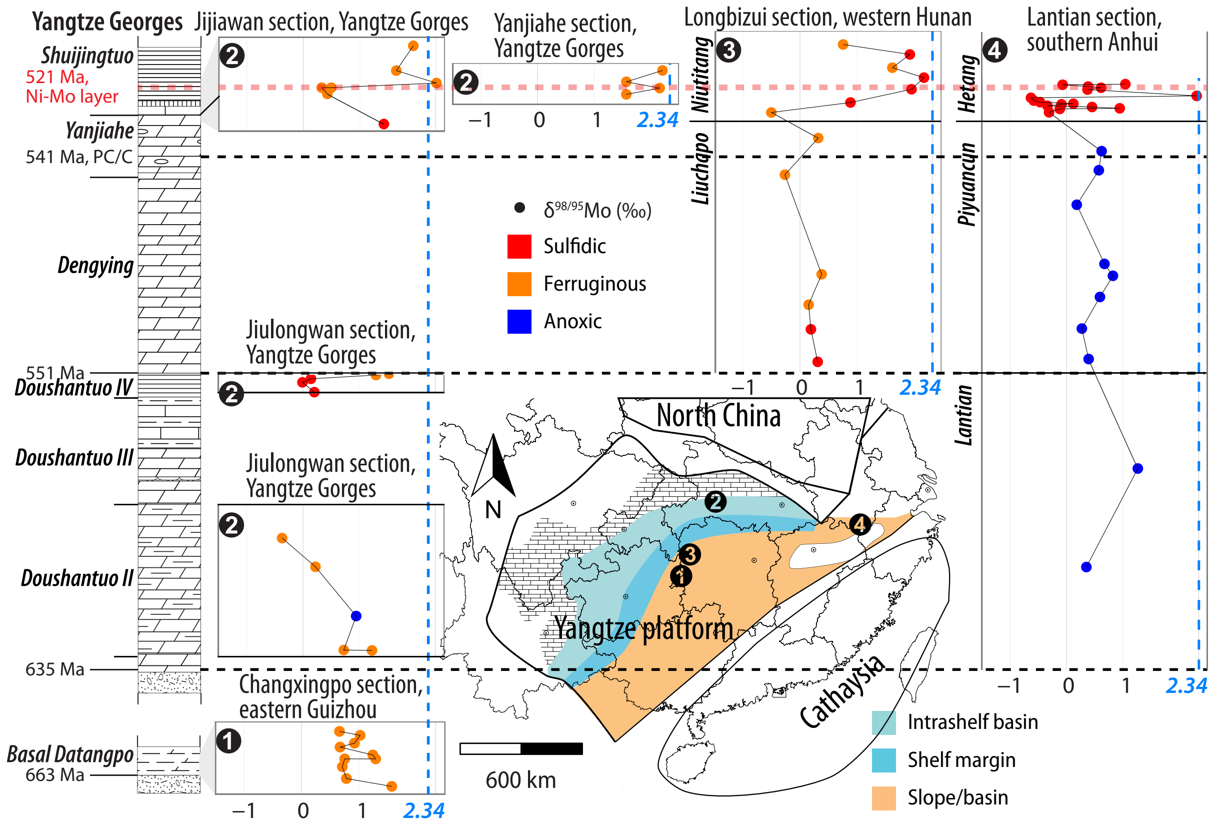

Supplementary Figure 2 | Molybdenum isotopic data from ca. 660 to 520 Ma on Yangtze Platform, South China. Circled numbers indicate sample sections: Jiulongwan (30°48'N, 111°04'E), Jijiawan (30°53'08"N, 110°52'03"E) and Yanjiahe (30°44'57"N, 111°02'08"E) sections in the Yangtze Gorges (intrashelf basin); Changxingpo (27°34'10"N, 109°23'08"E), Longbizui (28°30'15"N, 109°50'14"E) and Lantian sections (29°55'N, 118°05'E) (slope/basin). The color of the data points denotes local redox: sulphidic ($Fe_{Py}/Fe_{HR} > 0.7$, red) ferruginous ($Fe_{Py}/Fe_{HR} < 0.7$, orange) and anoxic ($Fe/Al > 0.5$, blue). The dashed blue lines mark the average $\delta^{98/95}$Mo value of modern seawater. The radii of the data points are greater than 2SE of each measurement.

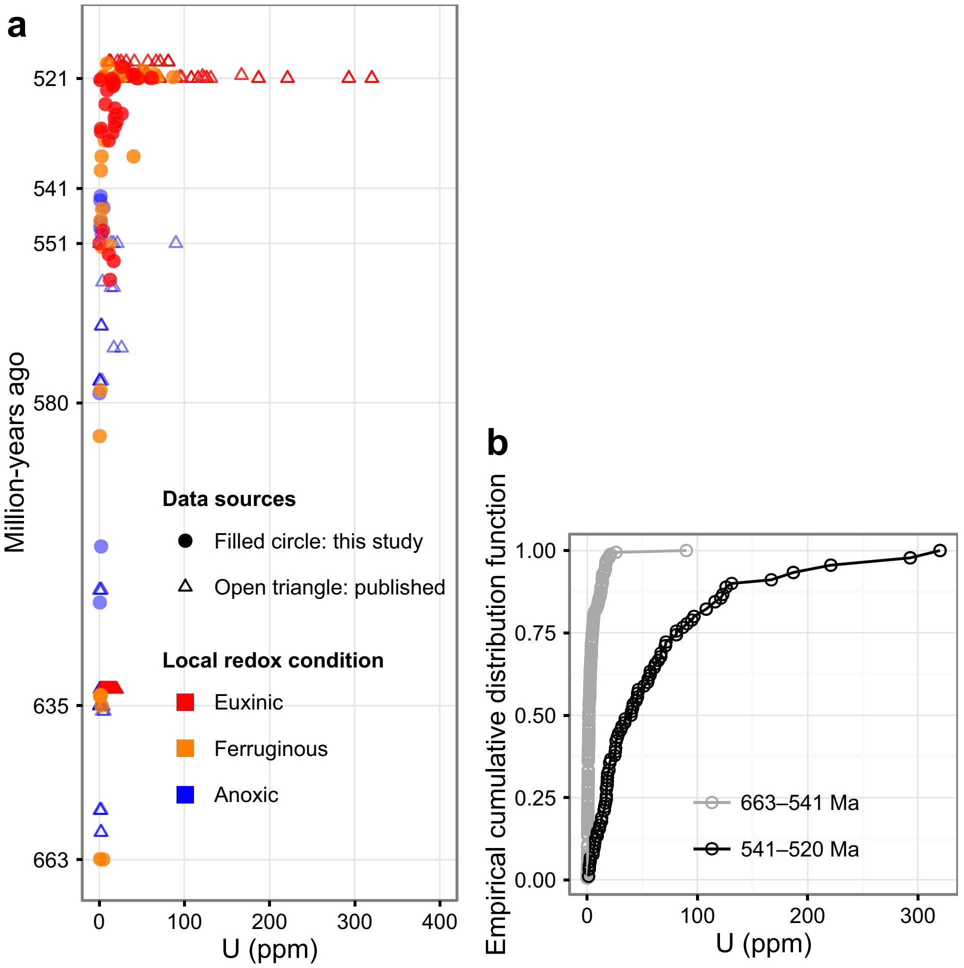

Supplementary Figure 3 | Compilation of U concentrations (a) from ca. 660 to 520 Ma and empirical cumulative density function of them (b). (**a**) The color of the data points denotes local redox. The shape of the data points denotes data sources. (**b**) The color of the data points denotes sample age groups.

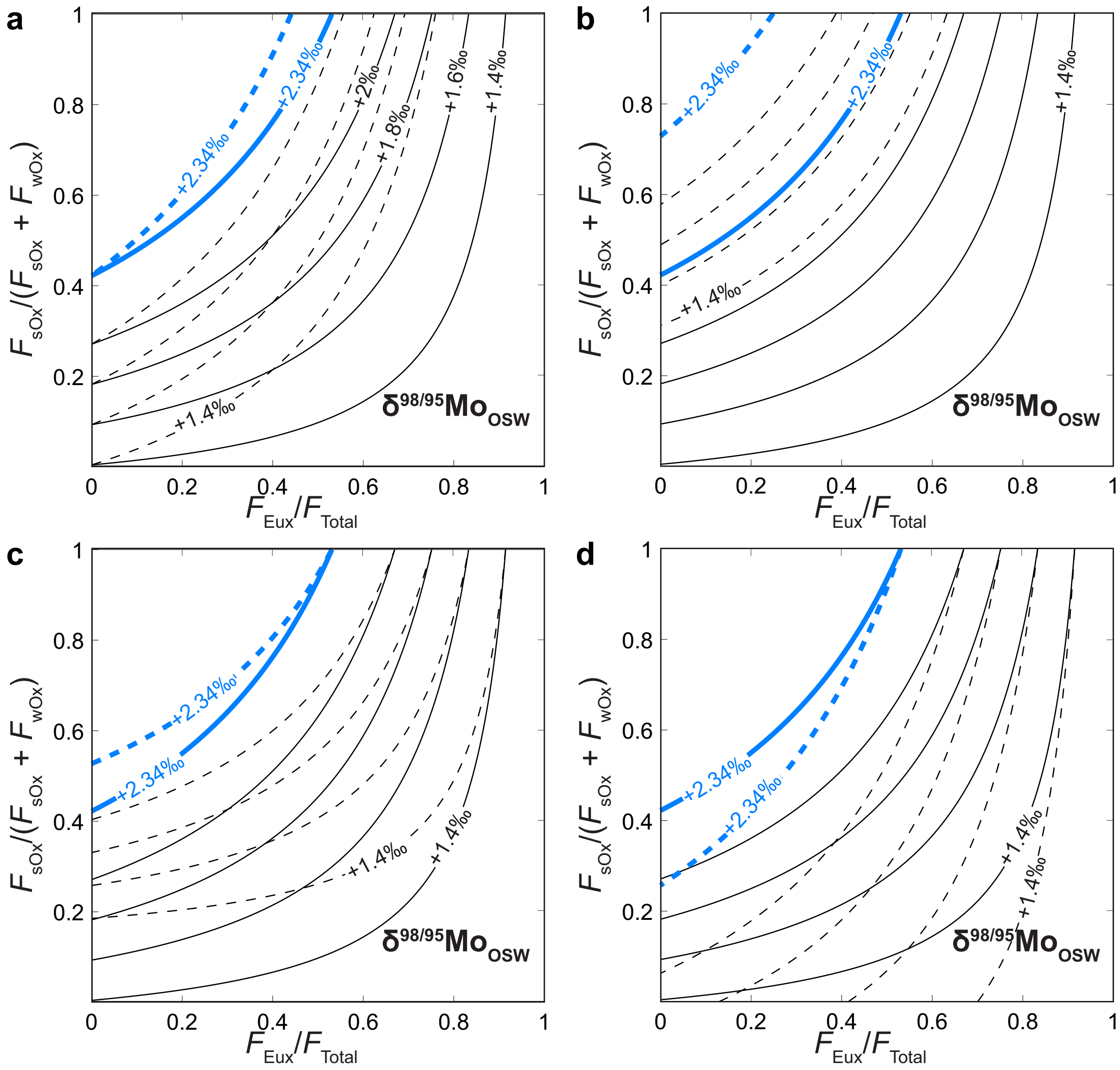

Supplementary Figure 4 | Results of sensitivity analysis. The solid curves are those depicted in the main text and Figure 2. Dashed curves in (**a**) are for $\Delta_{\mathrm{Eux-OSW}} = 0$‰, in (**b**) are for $\delta_{\mathrm{Rivers}} = 0$‰, in (**c**) are for $\Delta_{\mathrm{wOx-OSW}} = -0.2$‰ and in (**d**) are for $\Delta_{\mathrm{wOx-OSW}} = -1.2$‰.

Supplementary Table 1 | Parameters used in the model

$F_{\mathrm{River}} = 2.87 \times 10^{10}$ g yr^{-1}	Total Mo input flux per year [19]
$C_0 = 107$ nM	Mo concentration in modern open-ocean seawater [20]
$V_0 = 1.37 \times 10^{21}$ L	Total volume of the ocean
$A_{\mathrm{Total}} = 3.6 \times 10^{14}$ m^2	Total area of modern ocean
$F'_{\mathrm{Eux0}} = 4800 \times 10^{-6}$ g m^{-2} yr^{-1}	Modern rate of Mo output flux in euxinic settings [21]
$F'_{\mathrm{wOx0}} = 2700 \times 10^{-6}$ g m^{-2} yr^{-1}	Modern rate of Mo output flux in weakly oxic settings [21]
$F'_{\mathrm{sOx0}} = 40 \times 10^{-6}$ g m^{-2} yr^{-1}	Modern rate of Mo output flux in strongly oxic settings [22]
$\delta^{98/95}\mathrm{Mo}_{\mathrm{Rivers}} = +0.7‰$	Mo isotopic composition of modern rivers [23]
$\Delta_{\mathrm{Eux–OSW}} = -0.5‰$	Fractionation in euxinic settings [24]
$\Delta_{\mathrm{wOx–OSW}} = -0.7‰$	Fractionation in weakly oxic settings [25]
$\Delta_{\mathrm{sOx–OSW}} = -2.95‰$	Fractionation in strongly oxic settings [26]

Supplementary Note 1

Datangpo Formation

The interglacial Datangpo (equivalent to Xiangmeng) Formation is well developed in the border region of Hunan, Guizhou, and Sichuan provinces, South China. Glacial diamictite of the Gucheng (equivalent to Tiesiao or Chang'an) Formation equivalent to the Sturtian glacial sediment[27,28] is overlain by the ∼200-m-thick Datangpo Formation. The basal Datangpo Formation is marked by ∼1 m of black shale and 3–5 m of thin-bedded black Mn-carbonate or Mn-carbonate-bearing black shale, followed by ∼25-m-thick black or carbonaceous shale. The rest is grey shale and siltstone, which is overlain by the Nantuo diamictite equivalent to the Marinoan glacial sediment[27,29]. Zircons from an intercalated tuff bed in the basal Mn-carbonate of the Datangpo Formation are dated at 663 ± 4 Ma[27]. The Datangpo Formation in Hunan and Guizhou was likely deposited in a marginal basin with a connection to the open ocean along its southeast boundary[30,31].

Our samples were collected from the basal Datangpo Formation (within ∼2.2 m above the glacial diamictite) in an underground mining tunnel of Changxingpo Manganese Mine near the border of Guizhou and Hunan.

Doushantuo Formation

The Doushantuo Formation outcrops widely on the southern margin of the Yangtze platform in South China, with thickness varying from 40 to 260 m, and lateral facies changing from shallow-water phosphatic dolostone to deeper-water cherty dolostone and black shale[32]. In the Yangtze Gorges area (in Yichang, Hubei Province), the Doushantuo Formation is subdivided into four lithostratigraphic members[33]. Member I is a ∼5-m-thick cap dolostone, and overlies the Nantuo diamictite. An ash bed in lower Member I is dated at 635.2 ± 0.6 Ma[29]. Member II is characterised by ∼70 m of alternating organic-rich shale and dolostone beds with abundant pea-sized chert nodules. Zircons from an ash bed in lower Member II gave an age of 632.5 ± 0.5[29]. Member III is ∼70 m thick and is composed of dolostone and irregular chert bands in the lower part that passes up section into ribbon like interbedded limestone and dolostone. Member IV (also named Miaohe) is composed of an ∼10-m-thick black shale. This unit is widespread across the Yangtze Gorges area and defines the boundary between the Doushantuo Formation and the overlying Dengying Formation of terminal Neoproterozoic age. A tuff bed near the contact with the overlying Dengying Formation is dated at 551.1 ± 0.7 Ma[29].

Sedimentary facies analysis, sequence stratigraphic analysis and carbon isotope chemostratigraphy of sections in the Yangtze Gorges area suggest that the Doushantuo succession in this area was deposited in an offshore intra-shelf marine basin which was connected to the open ocean[34,35]. Fossils found in the richly fossiliferous Doushantuo Formation are summarised by McFadden *et al.*[35]. At the Jiulongwan section, complex large acanthomorph acritarchs, probable animal eggs and embryos, macroalgae, and filamentous and coccoidal cyanobacteria are preserved in chert nodules in member II. Member IV at the Miaohe section contains more than 20 species of macroscopic carbonaceous compression fossils, most of which are likely macroalgae[36], though a few may represent animals[37].

Our samples of the Doushantuo Formation were collected from Members II and IV of the Doushantuo Formation at the Jiulongwan section in the Yangtze Gorges area.

Lantian Formation

The Ediacaran System in southern Anhui consists of the Lantian Formation and overlying Piyuancun Formation, which are respectively correlated on the basis of regional lithostratigraphy with the Doushantuo and Dengying (551–541 Ma) Formations in the Yangtze Gorges area[38,39]. The Lantian Formation is divided into four members that can be correlated with the four divisions of the Doushantuo Formation[40]. The fossiliferous Lantian Member II is interpreted to have been deposited in open marine slope-basinal facies. Yuan *et al.*[40] reported a diverse assemblage of morphologically differentiated benthic macrofossils, preserved largely in situ as carbonaceous compressions, in Member II black shale of the Lantian Formation.

Our samples from the Lantian Formation are collected at the Lantian section.

Piyuancun and Dengying Formations

The Piyuancun Formation, located in southern Anhui, conformably overlies the Lantian Formation and consists mainly of black and grey chert beds. It is similar to the Liuchapo Formation in Hunan in

stratigraphic level and in lithology (see the following description for Liuchapo Formation). Both the Piyuancun and Liuchapo Formations, except for their upmost part, can be correlated with the Dengying Formation in the Yangtze Gorges area and eastern Yunnan (Fig. 1). Small shelly fossils (SSFs) are found in the uppermost parts of the equivalent Dengying Formation in the neighbouring Zhejiang Province[41], which indicates an Early Cambrian age for its topmost part[9].

Although the carbonate of Dengying Formation is not analyzed for Mo isotopes and Fe-speciation in this study, it will be described briefly here as it is important in stratigraphic correlation. In the Yangtze Gorges area, the Dengying Formation is composed of dolostone and limestone. It is conformably underlain by the Doushantuo Formation, and overlain by the Yanjiahe Formation. At the Yanjiahe and Jijiawan sections, the Dengying Formation is ~270 m thick and can be divided into three members, named Hamajing, Shibantan and Baimatuo in ascending order. The Dengying Formation yields frondose and fractal Ediacara fossils[42,43], large horizontal trace fossils made by bilaterian animals[44], and biomineralised metazoan tubular *Cloudina* fossils[45–47].

Our samples from the Piyuancun Formation are collected at the Lantian section.

Liuchapo Formation

The Liuchapo Formation in the Longbizui section, Guzhang County in western Hunan, is located in the deep-water lower slope to basinal setting[48]. It consists of dark grey to black chert, intercalated with thin layers of black shales. It is conformably underlain by the Doushantuo Formation and conformably overlain by the Niutitang Formation (see the following description for Niutitang Formation). The Liuchapo Formation can be correlated with the Piyuancun Formation at Lantian in southern Anhui and the shallow-water Dengying Formation in the Yangtze Gorges area. A tuff bed in the top of the Liuchapo Formation is dated at 536.3 ± 5.5 Ma[49], and the first appearance of the SSFs is in the upper Liuchapo chert[8]. Therefore, the Ediacaran–Cambrian boundary is placed at a level within the upper Liuchapo Formation[48](Fig. 1).

Our samples from the Liuchapo Formation are collected at the Longbizui section.

Yanjiahe and Zhujiaqing Formations

In the Yangtze Gorges area, the Yanjiahe Formation disconformably overlies the Dengying Formation, with an uneven contact boundary indicating a depositional hiatus (this study), and is itself overlain by the Shuijingtuo Formation with a disconformity[50]. The Yanjiahe Formation is composed of carbonate intercalated with chert (in lower and middle part) or black shale (upper part), and it can be roughly correlated to the Zhujiaqing Formation in eastern Yunnan, because of their similar SSF assemblages and zonations[50] as well as the carbon isotope evolution curve[51,52]. We sampled the top of it in two sections, Jijiawan and Yanjiahe, of Yangtze Gorges area.

Although early Cambrian sections in eastern Yunnan are not analyzed for Mo isotopes due to low Mo concentrations and lack of euxinic samples as indicated by Fe speciation data, their stratigraphy will be described here because of the stratigraphic continuity and well-developed biostratigraphy, and thus their importance in regional correlation (Fig. 1). A recent study[5], however, recognised a minor depositional hiatus between the Dengying and Zhujiaqing Formations and between the Zhujiaqing and Shiyantou Formations. The dolostone Baiyanshao Member of the Dengying Formation is overlain by the Zhujiaqing Formation. The Zhujiaqing Formation is subdivided into the Daibu (interbedded chert and dolostone), Zhongyicun (phosphorite-dominated) and Dahai (dolostone and limestone) members from the bottom upwards (Fig. 1). SSF assemblages I to III are successively found from lower Zhongyicun to Dahai members[4,5,53]. The overlying Shiyantou Formation comprises siltstone and shale, and contains the SSF assemblage IV in its upper part[53], which extends to the basal Yu'anshan Formation. The first appearance of the oldest trilobite *Parabadiella* occurs near the base of the Yu'anshan Formation[54,55]. The well persevered soft-bodied fossils of the Chengjiang Fauna is in the Yu'anshan Formation.

A SIMS U–Pb zircon age of 535.2 ± 1.7 Ma was obtained from the middle Zhongyicun Member (Bed 5) at the Meishucun section[15]. A SHRIMP U–Pb zircon age of 526.5 ± 1.1 Ma was obtained from the basal Shiyantou Formation[16]. Ni–Mo enrichments are observed in uppermost part of the Shiyantou Formation to the basal Yu'anshan Formation at the Xiaotan section, which can be correlated to the Ni–Mo sulphide layer in the Niutitang Formation in Guizhou and other areas[56]. The absolute time scale has not been established for the Yu'anshan Formation, but biostratigraphic correlation suggests it was deposited during Cambrian Stage 3[57–62].

Niutitang, Hetang, Shuijingtuo and Yu'anshan Formations

The Niutitang Formation, overlying the Denying Formation or Liuchapo Formation in Guizhou and Hunan provinces, consists mainly of black shale. In slope to basin sections, for example the Longbizui section in western Hunan, the Niutitang Formation conformably overlie the Liuchapo Formation and the boundary is transitional. The Niutitang Formation there consists of a lower unit of siliceous and phosphorous shale (~10 m thick) with thin phosphorite intercalations, and an upper thicker succession of black shale and mudstone[51]. In shallower-water settings, for example the Zhongnan section in Zunyi of northern Guizhou Province, the Niutitang Formation unconformably rests on the Dengying Formation. A few meters of the lowermost Niutitang Formation in this area is a condensed succession, and successively consists of stratiform cherts, nodular and bedded phosphorites and phosphate nodule-bearing carbonaceous black shales, a polymetallic Ni–Mo sulphide horizon, and black shales[17,63].

The Ni–Mo sulphide layer, with a typical thickness of 1 to 30 cm in Guizhou and northern Hunan[64], has varying Ni–Mo enrichment from several times higher than average upper continental crust contents to ore-level in different areas. It is a stratigraphic marker traceable in the black shale unit distributed in a NE-striking belt with a length of more than 1600 km along the southern margin of Yangtze platform[10]. The black shale unit containing this layer has different names in other areas, such as the Shuijingtuo Formation in the Yangtze Gorges, the Hetang Formation in Anhui and the Shiyantou Formation in Yunnan (see descriptions above). The genesis of the Ni–Mo enrichment is disputed. The candidates are a submarine hydrothermal venting origin[10,11,17,17,63,65,66] versus a synsedimentary seawater origin with very low terrigenous sedimentation rates in an anoxic environment[12,13,64,67,68], or a combination of both[14]. However, the consensus is that deposition of this layer was contemporary with sedimentation (Och *et al.*[56] and reference therein). Efforts have been made to date the layer[11,12,69–71], and recently a relatively precise composite Re–Os isochron age of 521 ± 5 Ma has been obtained from three mine sites in the Guizhou and Hunan provinces[18], which is consistent with the SHRIMP U–Pb zircon age of 532.3 ± 0.7 Ma obtained for a volcanic ash layer just beneath the sulphide ore layer and above the bottom Niutitang phosphorites at the Zhongnan section near Zunyi, Guizhou Province[17].

The lower part of the Niutitang Formation and its equivalents generally lack fossils[4], despite sponge spicules and helcionellid shells found in the lower Shiyantou Formation of Yunnan and the lower Niutitang Formation of Guizhou. Similar fossil assemblages within the Ni–Mo-enriched layer are found in the Sancha area of Zhangjiajie, Hunan Province and northern Guizhou Province (approximately 400 km away from Sancha)[10]. The upper part of the Niutitang Formation (and its equivalents, such as the Yu'anshan formation in Yunnan, the Shuijingtuo Formation in the Yangtze Gorges, the upper Hetang Formation in Anhui) contains fossils of Qiongzhusian age, such as the assemblage of hexactinellid sponges and demosponges, and some organic tissues and mineralised spicules in Hunan[72], and trilobites in Guizhou[73], Anhui[74] and Zhejiang[41]. Therefore, the upper Niutitang Formation is correlated approximately with Cambrian Stage 3[9,72].

Our samples of the Shuijingtuo Formation are collected from the Jijiawan and Yanjiahe sections, Yangtze Gorges area, Hubei; the samples of the Niutitang Formation are from the Longbizui section, western Hunnan; and the samples of the Hetang Formation are from the Lantian section, southern Anhui.

Supplementary References

1. Gradstein, F. M., Ogg, J. G., Schmitz, M. D. & Ogg, G. M. (eds.) *The Geologic Time Scale 2012* (Elsevier, 2012).

2. Qian, Y., Zhu, M., He, T. & Jiang, Z. New investigation of precambrian–cambrian boundary sections in eastern yunnan. *Acta Micropal. Sin.* **13**, 225–240 (1996).

3. Qian, Y., Li, G. & Zhu, M. The Meishucunian Stage and its small shelly fossil sequence in China. *Acta Palaeontol. Sin.* **40**, 54–62 (2001).

4. Steiner, M., Li, G., Qian, Y., Zhu, M. & Erdtmann, B.-D. Neoproterozoic to early Cambrian small shelly fossil assemblages and a revised biostratigraphic correlation of the Yangtze Platform (China). *Palaeogeogr., Palaeoclimatol., Palaeoecol.* **254**, 67–99 (2007).

5. Yang, B., Steiner, M., Li, G. & Keupp, H. Terreneuvian small shelly faunas of East Yunnan (South China) and their biostratigraphic implications. *Palaeogeogr., Palaeoclimatol., Palaeoecol.* **398**, 28–58 (2014).

6. Xiao, S., Hu, J., Yuan, X., Parsley, R. L. & Cao, R. Articulated sponges from the Lower Cambrian Hetang Formation in southern Anhui, South China: their age and implications for the early evolution of sponges. *Palaeogeogr., Palaeoclimatol., Palaeoecol.* **220**, 89–117 (2005).

7. Zhu, M. *et al.* Early Cambrian stratigraphy of east Yunnan, southwestern China: A Synthesis. *Acta Palaeontol. Sin.* **40**, 4–39 (2001).

8. Qian, Y. & Yin, G. Small shelly fossils from the lowermost Cambrian in Guizhou. *Prof. Pap. Stratigr. Palaeontol.* **13**, 91–123 (1984).

9. Yuan, X. *et al.* Towering sponges in an Early Cambrian Lagerstätte: Disparity between nonbilaterian and bilaterian epifaunal tierers at the Neoproterozoic-Cambrian transition. *Geology* **30**, 363–366 (2002).

10. Steiner, M., Wallis, E., Erdtmann, B.-D., Zhao, Y. & Yang, R. Submarine-hydrothermal exhalative ore layers in black shales from South China and associated fossils — insights into a Lower Cambrian facies and bio-evolution. *Palaeogeogr., Palaeoclimatol., Palaeoecol.* **169**, 165–191 (2001).

11. Jiang, S.-Y. *et al.* Trace- and rare-earth element geochemistry and Pb–Pb dating of black shales and intercalated Ni–Mo–PGE–Au sulfide ores in Lower Cambrian strata, Yangtze Platform, South China. *Miner. Deposita* **41**, 453–467 (2006).

12. Mao, J. *et al.* Re–Os dating of polymetallic Ni-Mo-PGE-Au mineralization in lower Cambrian black shales of South China and its geologic significance. *Econ. Geol.* **97**, 1051–1061 (2002).

13. Lehmann, B. *et al.* Highly metalliferous carbonaceous shale and Early Cambrian seawater. *Geology* **35**, 403–406 (2007).

14. Pašava, J. *et al.* Multiple sources of metals of mineralization in Lower Cambrian black shales of South China: Evidence from geochemical and petrographic study. *Resour. Geol.* **58**, 25–42 (2008).

15. Zhu, R. *et al.* SIMS U–Pb zircon age of a tuff layer in the Meishucun section, Yunnan, southwest China: Constraint on the age of the Precambrian-Cambrian boundary. *Sci. China, Ser. D: Earth Sci.* **52**, 1385–1392 (2009).

16. Compston, W., Zhang, Z., Cooper, J. A., Ma, G. & Jenkins, R. J. F. Further SHRIMP geochronology on the Early Cambrian of South China. *Am. J. Sci.* **308**, 399–420 (2008).

17. Jiang, S.-Y. *et al.* Early Cambrian ocean anoxia in South China. *Nature* **459**, E5–E6 (2009).

18. Xu, L., Lehmann, B., Mao, J., Qu, W. & Du, A. Re–Os age of polymetallic Ni-Mo-PGE-Au mineralization in Early Cambrian black shales of South China—A reassessment. *Econ. Geol.* **106**, 511–522 (2011).

19. Miller, C. A., Peucker-Ehrenbrink, B., Walker, B. D. & Marcantonio, F. Re-assessing the surface cycling of molybdenum and rhenium. *Geochim. Cosmochim. Acta* **75**, 7146–7179 (2011).

20. Collier, R. W. Molybdenum in the northeast Pacific ocean. *Limnol. Oceanogr.* **30**, 1351–1354 (1985).

21. Reinhard, C. T. *et al.* Proterozoic ocean redox and biogeochemical stasis. *Proc. Natl. Acad. Sci. U.S.A.* **110**, 5357–5362 (2013).

22. Scott, C. *et al.* Tracing the stepwise oxygenation of the Proterozoic ocean. *Nature* **452**, 456–459 (2008).

23. Archer, C. & Vance, D. The isotopic signature of the global riverine molybdenum flux and anoxia in the ancient oceans. *Nat. Geosci.* **1**, 597–600 (2008).

24. Dahl, T. W. *et al.* Devonian rise in atmospheric oxygen correlated to the radiations of terrestrial plants and large predatory fish. *Proc. Natl. Acad. Sci. U.S.A.* **107**, 17911–17915 (2010).

25. Poulson Brucker, R. L., McManus, J., Severmann, S. & Berelson, W. M. Molybdenum behavior during early diagenesis: Insights from Mo isotopes. *Geochem., Geophys., Geosyst.* **10** (2009).

26. Barling, J., Arnold, G. L. & Anbar, A. D. Natural mass-dependent variations in the isotopic composition of molybdenum. *Earth Planet. Sci. Lett.* **193**, 447–457 (2001).

27. Zhou, C. *et al.* New constraints on the ages of Neoproterozoic glaciations in south China. *Geology* **32**, 437–440 (2004).

28. Zhang, Q.-R., Li, X.-H., Feng, L.-J., Huang, J. & Song, B. A new age constraint on the onset of the Neoproterozoic glaciations in the Yangtze Platform, South China. *J. Geol.* **116**, 423–429 (2008).

29. Condon, D. *et al.* U–Pb ages from the Neoproterozoic Doushantuo Formation, China. *Science* **308**, 95–98 (2005).

30. Wang, J. & Li, Z.-X. History of Neoproterozoic rift basins in South China: implications for Rodinia break-up. *Precambrian Res.* **122**, 141–158 (2003).

31. Li, C. *et al.* Evidence for a redox stratified Cryogenian marine basin, Datangpo Formation, South China. *Earth Planet. Sci. Lett.* **331–332**, 246–256 (2012).

32. Zhou, C. & Xiao, S. Ediacaran δ^{13}C Chemostratigraphy of South China. *Chem. Geol.* **237**, 89–108 (2007).

33. Wang, X., Erdtmann, B. D., Chen, X. & Mao, X. Integrated sequence-, bio- and chemo- stratigraphy of the terminal Proterozoic to Lowermost Cambrian "black rock series" from central South China. *Episodes* **21**, 178–189 (1998).

34. Zhu, M. *et al.* Carbon isotope chemostratigraphy and sedimentary facies evolution of the Ediacaran Doushantuo Formation in western Hubei, South China. *Precambrian Res.* **225**, 7–28 (2013).

35. McFadden, K. A. *et al.* Pulsed oxidation and biological evolution in the Ediacaran Doushantuo Formation. *Proc. Natl. Acad. Sci. U.S.A.* **105**, 3197–3202 (2008).

36. Xiao, S., Yuan, X., Steiner, M. & Knoll, A. H. Macroscopic Carbonaceous Compressions in a Terminal Proterozoic Shale: A Systematic Reassessment of the Miaohe Biota, South China. *J. Paleontol.* **76**, 347–376 (2002).

37. Ding, L. *et al.* *Sinian Miaohe Biota of China* (Geological Publishing House, Beijing, 1996).

38. Yuan, X., Li, J. & Cao, R. A diverse metaphyte assemblage from the Neoproterozoic black shales of South China. *Lethaia* **32**, 143–155 (1999).

39. Yuan, X., Xiao, S., Li, J., Yin, L. & Cao, R. Pyritized chuarids with excystment structures from the late Neoproterozoic Lantian formation in Anhui, South China. *Precambrian Res.* **107**, 253–263 (2001).

40. Yuan, X., Chen, Z., Xiao, S., Zhou, C. & Hua, H. An early Ediacaran assemblage of macroscopic and morphologically differentiated eukaryotes. *Nature* **470**, 390–393 (2011).

41. He, S. & Yu, G. The small shelly fossils from the Palaeocambrian Meishucunian stage in western Zhejiang. *Geol. Zhejiang* **8**, 1–7 (1992).

42. Sun, W. Late Precambrian pennatulids (sea pens) from the Eastern Yangtze Gorge, China: Paracharnia gen. nov. *Precambrian Res.* **31**, 361–375 (1986).

43. Xiao, S., Shen, B., Zhou, C., Xie, G. & Yuan, X. A uniquely preserved Ediacaran fossil with direct evidence for a quilted bodyplan. *Proc. Natl. Acad. Sci. U.S.A.* **102**, 10227–10232 (2005).

44. Weber, B., Steiner, M. & Zhu, M.-Y. Precambrian–Cambrian trace fossils from the Yangtze Platform (South China) and the early evolution of bilaterian lifestyles. *Palaeogeogr., Palaeoclimatol., Palaeoecol.* **254**, 328–349 (2007).

45. Conway Morris, S., Mattes, B. W. & Menge, C. The early skeletal organism *Cloudina*: New occurrences from Oman and possibly China. *Am. J. Sci.* **290A** (1990).

46. Bengtson, S. & Zhao, Y. Predatorial borings in Late Precambrian mineralized exoskeletons. *Science* **257**, 367–369 (1992).

47. Chen, Z., Bengtson, S., Zhou, C. M., Hua, H. & Yue, Z. Tube structure and original composition of *Sinotubulites*: shelly fossils from the late Neoproterozoic in southern Shaanxi, China. *Lethaia* **41**, 37–45 (2008).

48. Wang, J., Chen, D., Yan, D., Wei, H. & Xiang, L. Evolution from an anoxic to oxic deep ocean during the Ediacaran–Cambrian transition and implications for bioradiation. *Chem. Geol.* **306–307**, 129–138 (2012).

49. Chen, D., Wang, J., Qing, H., Yan, D. & Li, R. Hydrothermal venting activities in the Early Cambrian, South China: Petrological, geochronological and stable isotopic constraints. *Chem. Geol.* **258**, 168–181 (2009).

50. Guo, J., Li, Y. & Li, G. Small shelly fossils from the early Cambrian Yanjiahe Formation, Yichang, Hubei, China. *Gondwana Res.* **25**, 999–1007 (2014).

51. Wang, D., Ling, H.-F., Li, D. & Chen, X. Carbon isotope stratigraphy of Yanjiahe Formation across the Ediacaran–Cambrian boundary in the area of Three Gorges. *J. Stratigr.* **36**, 21–30 (2012).

52. Li, D. *et al.* New carbon isotope stratigraphy of the Ediacaran–Cambrian boundary interval from SW China: implications for global correlation. *Geol. Mag.* **146**, 465–484 (2009).

53. Li, G. & Xiao, S. Tannuolina and Micrina (Tannuolinidae) From the Lower Cambrian of Eastern Yunnan, South China, and Their Scleritome Reconstruction. *J. Paleontol.* **78**, 900–913 (2004).

54. Hou, X. & Bergström, J. *Arthropods of the Lower Cambrian Chengjiang fauna, southwest China.* 45 (Wiley-Blackwell, 1997).

55. Hou, X.-G. *et al.* (eds.) *The Cambrian Fossils of Chengjiang, China: The Flowering of Early Animal Life* (Blackwell Publishing, 2004).

56. Och, L. M. *et al.* Redox changes in Early Cambrian black shales at Xiaotan section, Yunnan Province, South China. *Precambrian Res.* **225** (2013).

57. Zang, W. L. Sinian and Early Cambrian floras and biostratigraphy on the South China Platform. *Palaeontog. Abt. B Palaeophyt.* **224**, 75–119 (1992).

58. Zhang, J., Li, G., Zhou, C., Zhu, M. & Yu, Z. Carbon isotope profiles and their correlation across the Neoproterozoic–Cambrian boundary interval on the Yangtze Platform, China. *Bull. Natl. Mus. Nat. Sci.* **10** (1997).

59. Zhu, M., Zhang, J. & Li, G. Sedimentary environments of the Early Cambrian Chengjiang Biota: Sedimentology of the Yu'anshan Formation in Chengjiang County, eastern Yunnan. *Acta Palaeontol. Sin.* **40**, 80–105 (2001).

60. Steiner, M., Zhu, M.-Y., Weber, B. & Geyer, G. The Lower Cambrian of eastern Yunnan: Trilobite-based biostratigraphy and related faunas. *Acta Palaeontol. Sin.* **40**, 63–79 (2001).

61. Babcock, L. E., Zhang, W. & Leslie, S. A. The Chengjiang Biota: Record of the Early Cambrian diversification of life and clues to exceptional preservation of fossils. *GSA Today* **11** (2001).

62. Jenkins, R. J. F., Cooper, J. A. & Compston, W. Age and biostratigraphy of Early Cambrian tuffs from SE Australia and southern China. *J. Geol. Soc., London* **159**, 645–658 (2002).

63. Jiang, S.-Y. *et al.* Extreme enrichment of polymetallic Ni–Mo–PGE–Au in Lower Cambrian black shales of South China: An Os isotope and PGE geochemical investigation. *Palaeogeogr., Palaeoclimatol., Palaeoecol.* **254**, 217–228 (2007).

64. Xu, L., Lehmann, B. & Mao, J. Seawater contribution to polymetallic Ni-Mo-PGE-Au mineralization in Early Cambrian black shales of South China: Evidence from Mo isotope, PGE, trace element, and REE geochemistry. *Ore Geol. Rev.* **52**, 66–84 (2013).

65. Lott, D. A., Coveney, R. M., Murowchick, J. B. & Grauch, R. I. Sedimentary exhalative nickel-molybdenum ores in South China. *Econ. Geol.* **94**, 1051–1066 (1999).

66. Jiang, S.-Y., Zhao, K.-D., Li, L., Ling, H.-F. & Zhu, M. Highly metalliferous carbonaceous shale and Early Cambrian seawater: COMMENT. *Geology* **35**, e158–e159 (2007).

67. Lehmann, B., Mao, J., Li, S., Zhang, G. & Zeng, M. Re–Os dating of polymetallic Ni-Mo-PGE-Au mineralization in lower Cambrian black shales of South China and its geologic significance—A Reply. *Econ. Geol.* **97**, 1051–1061 (2002).

68. Lehmann, B., Nägler, T. F., Wille, M., Holland, H. D. & Mao, J. Highly metalliferous carbonaceous shale and Early Cambrian seawater: REPLY. *Geology* **35**, e159 (2007).

69. Horan, M. F. *et al.* Rhenium and osmium isotopes in black shales and Ni-Mo-PGE-rich sulfide layers, Yukon Territory, Canada, and Hunan and Guizhou provinces, China. *Geochim. Cosmochim. Acta* **58**, 257–265 (1994).

70. Li, S. *et al.* Rhenium-osmium isotope constraints on the age and source of the platinum mineralization in the Lower Cambrian black rock series of Hunan-Guizhou provinces, China. *Sci. China, Ser. D: Earth Sci.* **46**, 919–927 (2003).

71. Jiang, S. *et al.* Re–Os isotopes and PGE geochemistry of black shales and intercalated Ni-Mo polymetallic sulfide bed from the Lower Cambrian Niutitang Formation, South China. *Prog. Nat. Sci.* **13**, 788–794 (2003).

72. Steiner, M., Zhu, M., Zhao, Y. & Erdtmann, B.-D. Lower Cambrian Burgess Shale-type fossil associations of South China. *Palaeogeogr., Palaeoclimatol., Palaeoecol.* **220**, 129–152 (2005).

73. Yang, A., Zhu, M., Zhang, J. & Li, G. Early Cambrian eodiscoid trilobites of the Yangtze Platform and their stratigraphic implications. *Prog. Nat. Sci.* **13**, 861–866 (2003).

74. Li, C., He, J. & Ye, H. Discovery of Early Cambrian trilobites in Guichi of Anhui Province. *J. Stratigr.* **14** (1990).
